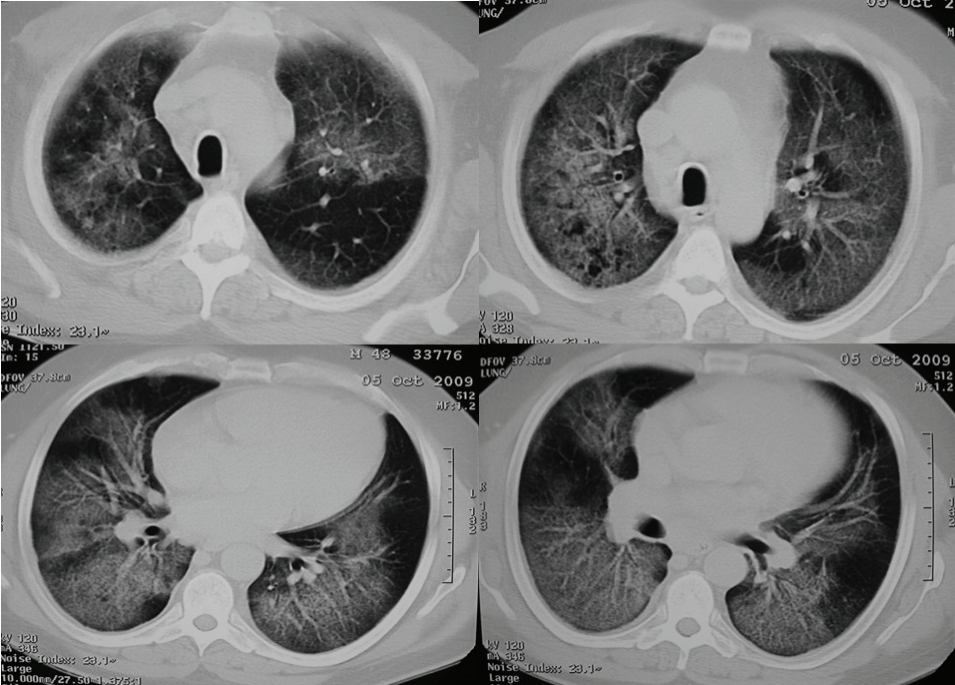# Bilateral Ground Glass Opacities and Acute Respiratory Failure: “Auribus Tenere Lupum”

**DOI:** 10.31138/mjr.33.4.465

**Published:** 2022-12-31

**Authors:** Ilias E. Dimeas, Sotirios I. Sinis, George E. Dimeas, Zoe Daniil

**Affiliations:** Department of Respiratory Medicine, Faculty of Medicine, University of Thessaly, Biopolis, Larissa, Greece

**Keywords:** bilateral ground glass opacities, systematic erythematosus lupus, diffuse alveolar haemorrhage

**Figure 1:**
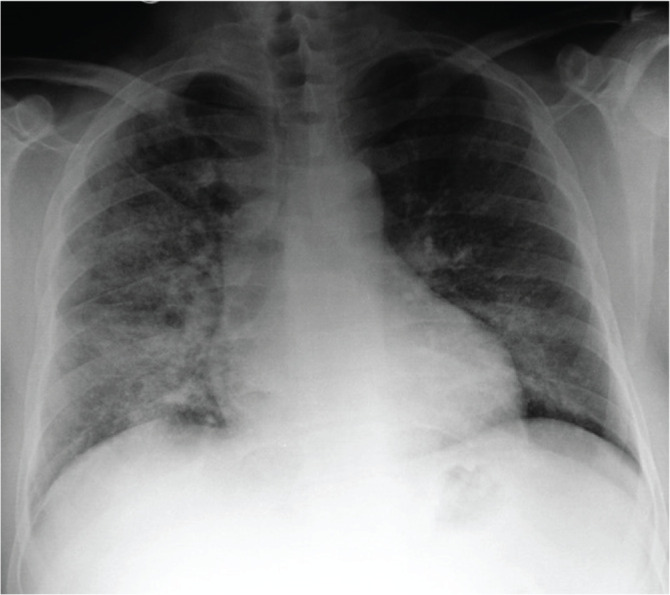


A 36-year-old man without any remarkable medical history visited the Emergency Department of our hospital reporting fever for the last 2 days and acute onset of dyspnoea since few hours ago. He did not complain of any chest pain, haemoptysis, or haematuria. During physical examination, bilateral crackles in both lung fields and tachycardia were noted. Hypoxemia requiring 3lt oxygen therapy via nasal cannula, mild anaemia of Hb=10 g/dL, low value natriuretic peptide, normal kidney function and a value of C-reactive protein at 7 mg/dL (<0.5 mg/dL) were found. An x-ray (**Figure 1**) revealed bilateral air space opacifications and an incipient consolidation in the right low lung field. With a provisional diagnosis of respiratory infection, ceftriaxone, moxifloxacin, and oseltamivir were administered to the patient and was then admitted to the Department of Respiratory Medicine. During the first hours, the patient deteriorated, so a computed tomography was performed (**Figure 2**), showing bilateral ground glass opacities consistent with acute respiratory distress syndrome. Unfortunately, due to acute respiratory failure nonresponding to high flow nasal cannula oxygen therapy, the patient was intubated and was then transferred to the intense care unit. With the patient sedated, a bronchoscopy was performed revealing haemorrhagic secretions from both lung with a bronchial lavage fluid result consisting of 38% haemosiderin-laden macrophages. The presence of ≥20% haemosiderin-laden macrophages in bronchial lavage fluid is the most commonly employed criterion for diffuse alveolar haemorrhage. High value of antinuclear antibodies (1/2560) combined with a rheumatologic consultation set the diagnosis of systematic lupus erythematosus (SLE) firstly appeared with diffuse alveolar haemorrhage or historically mentioned as acute lupus pneumonitis. “Acute lupus pneumonitis” term was commonly used by clinicians to define the ‘lupus’ patient presenting with fever and pulmonary infiltrates. However, this term refers to nothing specific since the differential diagnosis includes several acute respiratory conditions that occur in SLE. Respiratory manifestations most commonly occurring in SLE are pneumonias mainly infectious, pleural disease and pulmonary thromboembolism. Clinical entities regarding pulmonary vascular disease consist in pulmonary arterial hypertension, diffuse alveolar haemorrhage, vasculitis, and chronic thromboembolic pulmonary hypertension. A special effort should be made to assure diagnosis and initiate targeted treatment.

**Figure 2:**